# Treatment patterns and impact of glucocorticoids on health outcomes in generalized myasthenia gravis: a retrospective observational study based on the Medical Data Vision database in Japan

**DOI:** 10.3389/fneur.2025.1625457

**Published:** 2025-10-10

**Authors:** Hirofumi Teranishi, Koichi Tsuda, Daisuke Harada, Challa Yachendra, Anthony Nguyen, Mai Sato, Masanori P. Takahashi, Cécile Blein

**Affiliations:** ^1^argenx Japan, Tokyo, Japan; ^2^ZS Associates, Bengaluru, India; ^3^ZS Associates, Durham, NC, United States; ^4^ZS Associates, New York, NY, United States; ^5^Osaka University, Suita, Japan; ^6^argenx BVBA, Ghent, Belgium

**Keywords:** corticosteroids, intravenous immunoglobulin, myasthenia gravis, real-world evidence, osteoporosis, early fast-acting treatment, steroid-sparing

## Abstract

**Introduction:**

Generalized myasthenia gravis (gMG) is a rare, chronic, autoimmune disorder with a substantial disease burden. Oral glucocorticoids (GCs) are one of the common early-line treatments used globally, but there are growing concerns regarding risks associated with GC adverse effects, which can have serious medical consequences. In Japan, an important gMG treatment goal is minimal disease manifestation with a low target GC dose (≤5 mg/day) to minimize GC exposure. This study aimed to assess gMG treatment patterns in Japan, with a focus on GC exposure and incident comorbidities associated with long-term GC use.

**Methods:**

This was a retrospective, observational database study of patient data from the Medical Data Vision (MDV) database (2008–2022) in Japan. Adults (aged ≥18 years) with ≥2 gMG diagnosis records were considered. First gMG diagnosis (ocular MG excluded) between 2018 and 2021 was considered the index date. All available follow-up was considered if the patient had ≥1 activity in the database annually after the index date.

**Results:**

Of 9,687 patients with gMG (mean age: 65 years; 56% female; follow-up period: 3.2 years) included in the study, 3,696 (38.2%) were newly diagnosed (2018 onwards) and 5,991 (61.8%) were previously diagnosed (before 2018). Acetylcholinesterase inhibitor (AChEi) and GC monotherapy were the most common initial therapies after index, and most patients were treated with combinations of AChEi, GC, and/or nonsteroidal immunosuppressive therapies (NSISTs). The average daily GC dose was 8.5 mg/day (newly diagnosed: 10.6 mg/day; previously diagnosed: 7.5 mg/day). The target daily dose of 5 mg/day was exceeded by 70% of GC-treated patients, and 26% exceeded 10 mg/day (newly diagnosed: 35%; previously diagnosed: 21%). Compared to patients with no GC exposure, GC use was dose-dependently associated with osteoporosis, thrombosis, diabetes, and hyperlipidemia/hypercholesterolemia, even at GC doses ≤5 mg/day.

**Conclusion:**

Most patients were treated with AChEi, GC, or NSIST monotherapy or combination therapies and received GCs exceeding 5 mg/day, which was associated with developing several GC-associated comorbidities in a dose-dependent manner. To achieve treatment goals, patients with gMG may benefit from additional treatment approaches to reduce GC usage.

## 1 Introduction

Generalized myasthenia gravis (gMG) is a rare, chronic, autoimmune disorder that predominantly manifests as generalized skeletal muscle weakness and exercise induced weakness (1–4). The pathophysiology of gMG involves autoantibodies that impair neuromuscular transmission, trigger complement-mediated damage, and block normal acetylcholine receptor signaling (1–3). Patients with gMG frequently endure a substantial disease burden, accompanied by negative impact on health-related quality of life (HRQoL) (5–7). This impact arises not only from the primary disease but also from its associated comorbidities and treatment-related challenges (5–7). Consistent with the overall rising prevalence of autoimmune diseases, the global prevalence of gMG also appears to be increasing (8, 9). A recent systematic review revealed that the mean prevalence and incidence of gMG have more than doubled over the past 7 decades (10). In line with global trends, the prevalence of diagnosed gMG in Japan has doubled from 2006 to 2014, reaching 23.1 cases per 100,000 (11, 12). In the United States (US), the diagnosed prevalence of gMG in 2021 was 37.0 per 100,000 persons, with an incidence rate of 3.1 per 100,000 persons per year (12).

According to the international consensus guidelines for gMG, treatment typically begins with acetylcholinesterase inhibitors (AChEis) and/or immunosuppressive therapies, primarily oral glucocorticoids (GCs) or non-steroidal immunosuppressive therapies (NSISTs) (13, 14). Thymectomy is advised for patients diagnosed with thymoma (13, 14). Intravenous (IV) or subcutaneous (SC) immunoglobulin (Ig) and plasma exchange (PLEX) are commonly used as rescue treatment during exacerbations or myasthenic crisis and are also used chronically in patients with inadequate symptom control (13, 14). Moreover, the approval of novel targeted therapies (biologics) for gMG in recent years is rapidly transforming the gMG treatment landscape, offering new avenues for targeted and effective disease management (15). While most global regions adhere to the international consensus guidelines for gMG treatment, Japan has specific gMG treatment guidelines, most recently updated in 2022 (16, 17).

Although the focus on symptom management is consistent across guidelines, treatment goals in Japan specifically include maintenance of HRQoL by addressing both mental and physical well-being as specific treatment goals (13, 14, 16). Mental and physical health is associated with both minimization of disease manifestations and low dose of GC (7). Therefore, the primary goal of MG treatment in the Japanese guidelines is to achieve minimal disease manifestation as fast as possible, with oral GC not exceeding 5 mg/day (16). To achieve this goal, an early fast-acting treatment (EFT) strategy is recommended. In this strategy, early use of fast-acting treatment, such as IVIg, PLEX, intravenous methylprednisolone (IVMP), and/or immunoadsorption plasmapheresis (IAPP) is recommended, particularly when oral therapies (low dose of AChEi, GC, and NSIST) elicit insufficient response, instead of increasing the dose of GC above the recommended ≤10 mg/day initially (18). Patients treated according to the EFT strategy have achieved minimal disease manifestation with GC ≤ 5 mg at a higher rate compared to patients without EFT (19). High-dose oral GC regimens with escalation and de-escalation schedules are not recommended due to their association with side effects, decreased HRQoL, and lack of evidence for significant benefits in achieving complete remission or early minimal disease manifestation (16). The Japanese guidelines represent a strong commitment to not only provide rapid symptom control but also aggressively reduce GC usage among patients with gMG. Of note, the 2025 update of the Japanese guidelines expanded the list of recommended biologics (initially included only eculizumab and rituximab) for patients with inadequate clinical response to include the complement C5 inhibitors zilucoplan and ravulizumab and the neonatal Fc receptor (FcRn) blockers efgartigimod and rozanolixizumab. However, evidence is limited, and further data are still needed (20).

Although GCs are generally effective at controlling symptoms in autoimmune diseases such as gMG, long-term GC exposure is associated with potentially serious comorbidities such as hypertension, bone fractures, cataracts, gastrointestinal conditions, and metabolic conditions such as hyperglycemia, type 2 diabetes, and weight gain (16, 21). GC-induced osteoporosis is a major concern, accounting for up to 25% of adverse events (AEs) associated with GC therapy (22). It is estimated to be the underlying cause of 30–50% of fractures observed in patients undergoing GC treatment (22). Reducing or eliminating GC usage is likely to positively impact patients with gMG by reducing the risk and severity of AEs-associated with long-term GC use (23, 24).

While the impact of these treatment recommendations on real-world GC exposure among patients with gMG in Japan is of high interest, a limited number of large-scale studies in gMG have been conducted in Japan (4–6, 25, 26). The digital observational study MyRealWorld MG reported a high disease burden in patients with gMG in a multicountry cohort that includes patients from Japan (5, 6). A 2022 non-interventional survey conducted in Japan in patients with gMG revealed that 1 in 4 patients with gMG was dissatisfied with life, indicating multiple unmet needs in the gMG patient population (4). Although these studies provide important insights into the disease burden of gMG in Japan, there is a paucity of data on GC utilization in clinical practice and its potential impact on the development of GC-associated comorbidities among patients with gMG in Japan. Therefore, this study was conducted with the aim to assess treatment patterns in patients with gMG and evaluate the impact of GC treatment on the development of comorbidities in patients with gMG living in Japan, to inform better treatment decision-making.

## 2 Methods

### 2.1 Study design and data source

This was a retrospective, observational study from April 1, 2008, to December 31, 2022, of patients diagnosed with gMG from the Medical Data Vision (MDV) database in Japan. The MDV database contains administrative data from over 540 hospitals, including the status and treatment of approximately 50 million patients in Japan (27). It is derived only from facilities included in the Diagnosis Procedure Combinations and is not linked to information from other hospitals (27). Only medicines prescribed at Diagnosis Procedure Combinations facilities were therefore included.

### 2.2 Study population

Among patients with ≥2 gMG diagnoses (disease codes 3580006, 8830896, 8846112, 8846113, 8846179, 3589004; ≥30 days apart) between 2008 and 2022, those with at least one gMG diagnosis between 2018 and 2021 were included, with the first gMG diagnosis between 2018 and 2021 as the index date. Disease codes specific to ocular MG were not considered. Patients who were aged ≥18 years at the index date and had at least one activity in the database during the 1 year before and 1 year after the index date were included in the study (Supplementary Figure 1). Patients with any of the exclusionary diagnosis claims (Supplementary Table 1) 1 year before or at any time after the index date were excluded.

### 2.3 Study variables and outcomes

Study variables included patient baseline characteristics such as age and sex at index and gMG treatments used 1 year pre-index, including AChEis, GCs, NSISTs (azathioprine, cyclophosphamide, cyclosporine, methotrexate, mycophenolate mofetil, and tacrolimus), biologics, IVIg, PLEX, and thymectomy. Charlson Comorbidity Index (CCI), a composite weighted index including 17 categories of diseases to predict mortality, was calculated based on comorbidities present in the 1 year pre-index (28). Study outcomes included yearly treatment patterns, GC exposure, and associations of comorbidities with GC exposure after index.

### 2.4 Analytical and statistical methods

Descriptive statistics were used to describe patient characteristics and treatment usage. As an exploratory exercise in the treatment usage analysis, IVIg claims were clustered into IVIg episodes if claims were within 5 days of each other. Patients who had ≥3 IVIg episodes in year 1 were considered frequent IVIg users for subgroup analyses. To calculate overall GC exposure, GC (prednisone equivalent) episodes were created by combining claims that occurred <28 days of each other (Supplementary Figure 2). The average daily dose for each patient was calculated as average per day over the period the patient was exposed to GC. To evaluate the association of GC exposure with comorbidity development within 1 year after index, GC usage was captured up to the development of the outcome of interest or 1 year post-index, whichever occurred earlier. Both binary (yes/no) and categorical (low [>0 to ≤5 mg/day], medium [>5 to ≤10 mg/day], and high [>10 mg/day] average daily GC dose) exposure definitions were analyzed. Incidence of gMG-related comorbidities and the impact of GC use on comorbidity occurrence were analyzed using hazard ratios (HRs) with 95% confidence intervals (CIs). For each comorbidity of interest, patients who had any claims including the comorbidity pre-index or within 6 months post-index were excluded from the analysis. Cox proportional hazards regression was used to estimate the incidence of each outcome adjusted for age, sex, CCI, disease duration, and other gMG treatments.

## 3 Results

### 3.1 Patient characteristics

**Figure 1:**
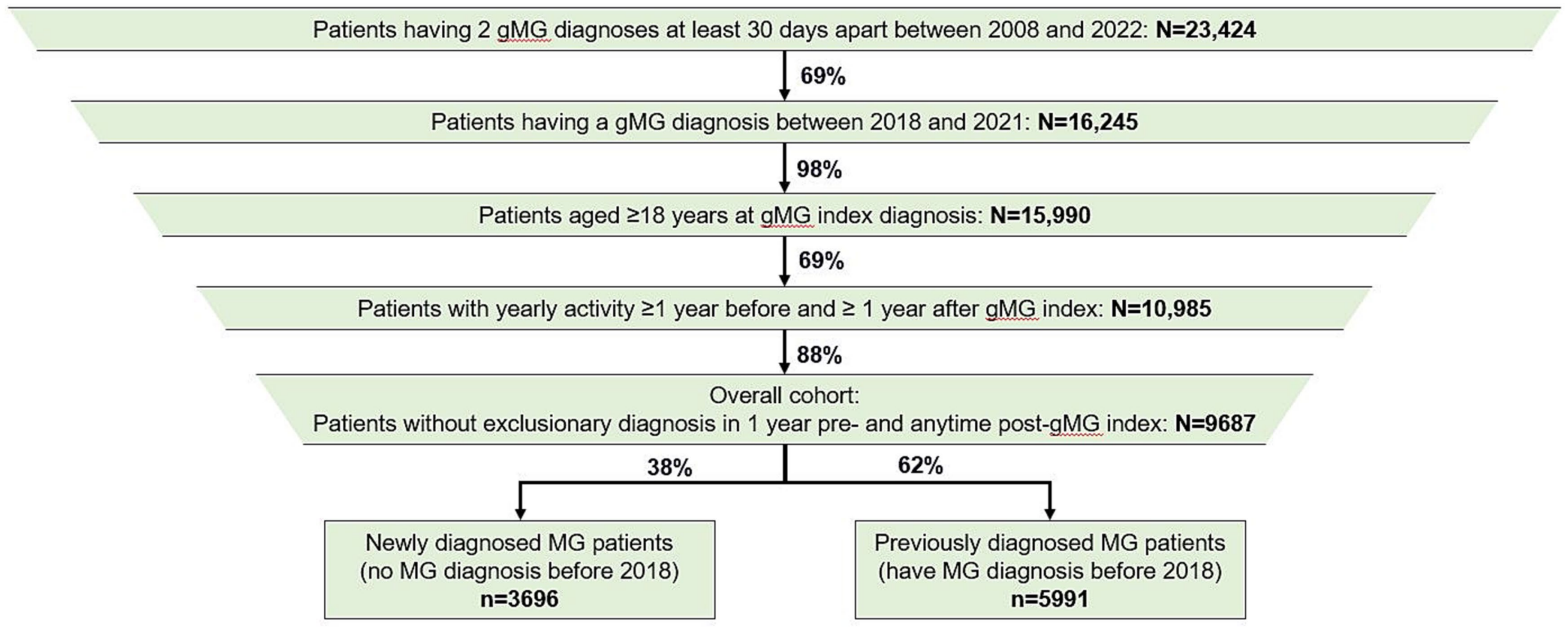
Patient disposition. gMG, generalized myasthenia gravis; MG, myasthenia gravis.

A total of 9,687 patients were included in the study, with a mean follow-up of 3.2 years (Figure 1 and Table 1). At baseline, mean (standard deviation [SD]) age was 65 (15.2) years, 56% were female, and mean CCI (excluding the index date) was 1.57. Of the 9,687 patients, 3,696 (38.2%) were newly diagnosed (i.e., did not have gMG diagnosis before 2018) and 5,991 (61.8%) were previously diagnosed (i.e., had gMG diagnosis before 2018).

**Table 1:** Baseline patient characteristics.

	Overall	Newly diagnosed	Previously diagnosed
	*N* = 9,687	*n* = 3,696	*n* = 5,991
Age			
Mean (SD), years	65 (15.2)	65 (15.2)	65 (15.2)
18–40 years, n (%)	704 (7)	267 (7)	437 (7)
41–65 years, *n* (%)	3,444 (36)	1,209 (33)	2,235 (37)
>65 years, *n* (%)	5,539 (57)	2,220 (60)	3,319 (55)
Sex, *n* (%)			
Male	4,221 (44)	1760 (48)	2,461 (41)
Female	5,466 (56)	1936 (52)	3,530 (59)
CCI score, mean (SD)			
Including claims on the index date^a^	1.72 (2)	1.84 (2.28)	1.66 (1.81)
Excluding claims on the index date^b^	1.57 (1.91)	1.48 (2.10)	1.62 (1.79)
Treatments 1 year pre-index of gMG diagnosis, *n* (%)^c^			
AChEi	3,135 (32)	262 (7)	2,873 (48)
GC	3,310 (34)	457 (12)	2,853 (48)
NSIST	2,169 (22)	190 (5)	1,979 (33)
Biologics	13 (0)	11 (0)	2 (0)
IVIg	307 (3)	66 (2)	241 (4)
PLEX	93 (1)	2 (0)	91 (2)
Thymectomy	68 (1)	5 (0)	63 (1)
Years of follow-up, mean (SD)	3.2 (1.6)	2.0 (1.3)	3.8 (1.4)

^a^CCI calculated using disease claims in the year before index date including index date. ^b^CCI calculated using disease claims in the year before index date excluding index date. ^c^Percentages may not add up to 100% as patients may have had multiple treatments. AChEi, acetylcholinesterase inhibitor; CCI, Charlson comorbidity index; GC, glucocorticoid; gMG, generalized myasthenia gravis; IVIg, intravenous immunoglobulin; NSIST, non-steroidal immunosuppressive therapy; PLEX, plasma exchange; SD, standard deviation.

### 3.2 Generalized myasthenia gravis treatment patterns

**Figure 2:**
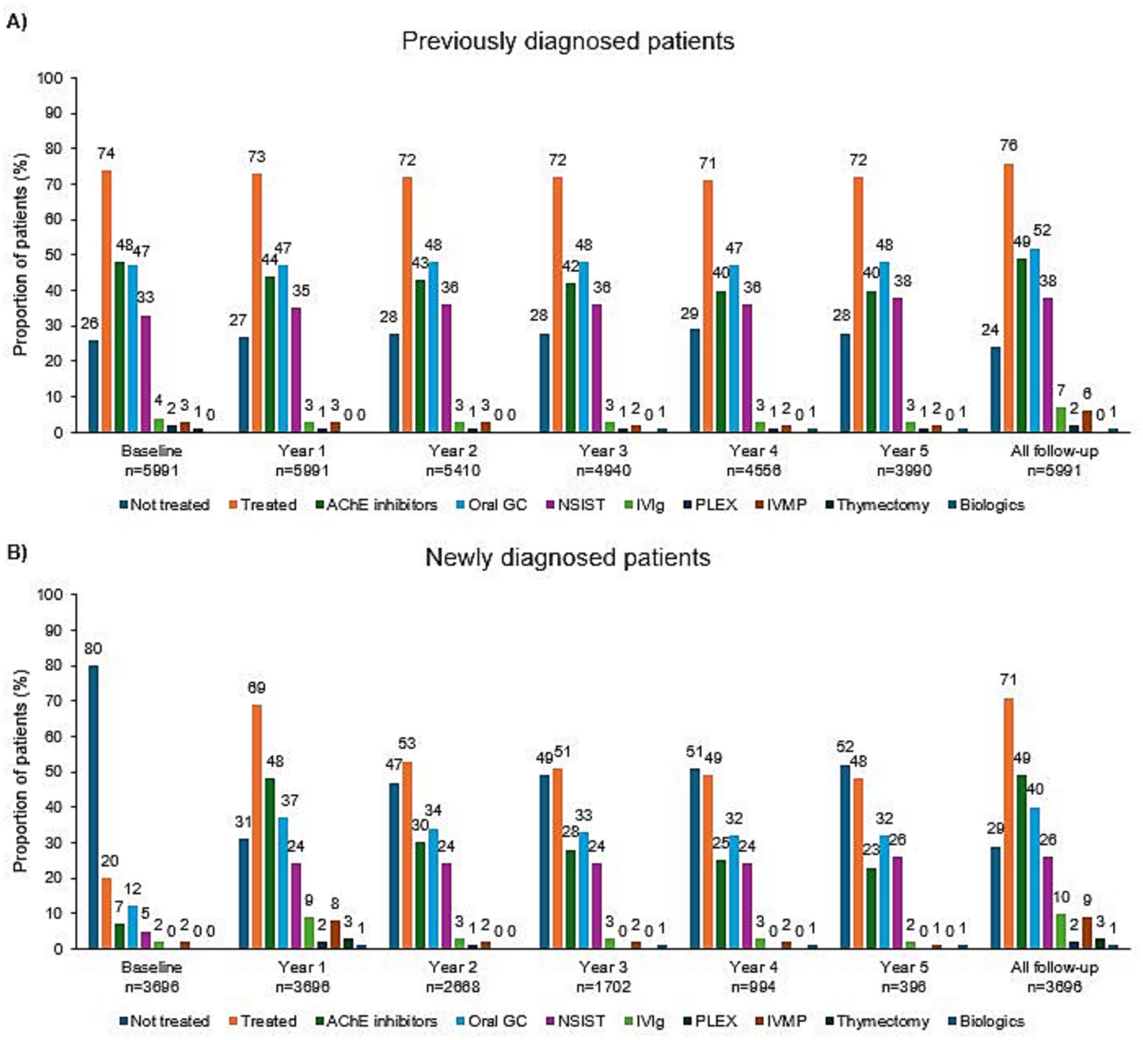
Year-by-year treatment utilization in **(A)** previously and **(B)** newly diagnosed patients with gMG. AChE, acetylcholinesterase; GC, glucocorticoid; gMG, generalized myasthenia gravis; IVIg, intravenous immunoglobulin; IVMP, intravenous methylprednisolone; NSIST, nonsteroidal immunosuppressive therapy; PLEX, plasma exchange.

Over a maximum follow-up period of 5 years post-index, the most frequently utilized treatments were AChEis, GCs, and NSISTs (Figure 2). In previously diagnosed patients with gMG, annual usage of treatments was relatively stable, consisting most commonly of AChEis, GCs, and NSISTs, with less use of other therapies (Figure 2A). In newly diagnosed patients with gMG, treatment utilization clearly changed from baseline to the first year after diagnosis. The percentage of patients treated with fast-acting treatments, such as IVIg, PLEX, and IVMP, was high in the first year in newly diagnosed patients and fluctuated in the first 2 years before stabilizing into treatment patterns similar to the patients in the previously diagnosed group (Figure 2B). Among a subgroup of 167 patients who used frequent IVIg (≥3 IVIg episodes) in the first year after index with follow-up data available, over half (59%, 33/56) used IVIg at least once yearly up to year 5 post-index (Supplementary Table 2). Additionally, concomitant usage of AChEis, GCs, and NSISTs also persisted among these patients up to year 5.

The treatment patterns analysis indicated that most patients were treated with a combination of AChEis, GCs, and/or NSISTs, with at least one treatment change or addition experienced by the majority (Table 2 and Supplementary Figures 3A–C). AChEi or GC monotherapy was most common as the first observed treatment after index. While most patients remained on this regimen until the end of follow-up, a substantial subset of patients switched treatment combinations up to their third observed therapy combination.

**Table 2:** Overall treatment patterns over the 5-year follow-up period.

Duration of treatment or combination	*N* = 9,687 patients
First treatment or combination after index	
Mean (SD), days	565 (652)
Median (IQR), days	240 (36–1,002)
Second treatment or combination after index	
Mean (SD), days	163 (325)
Median (IQR), days	30 (10–112)
Third treatment or combination after index	
Mean (SD), days	216 (351)
Median (IQR), days	49 (15–252)
**Patients who only used 1 treatment or combination, *n* (%)**	**3,399 (35)**
Treatment or combination used until discontinued or end of follow-up	
AChEi only	1,541 (45)
GC only	940 (28)
NSIST only	302 (9)
AChEi + GC	159 (5)
GC + NSIST	154 (5)
AChEi + GC + NSIST	135 (4)
AChEi + NSIST	128 (4)
Any advanced therapy	40 (1)
**Patients who switched treatment or combination only once, *n* (%)**	**3,746 (39)**
Top 5 most common changes	
AChEi → AChEi + GC	378 (10)
GC + NSIST → GC only	271 (7)
AChEi + GC → GC only	268 (7)
GC + NSIST → NSIST only	226 (6)
AChEi + GC → AChEi only	203 (5)
**Patients who switched treatment or combination twice or more, *n* (%)**	**2,992 (31)**
Top 5 most common changes	
GC + NSIST → GC only → GC + NSIST	225 (8)
AChEi + GC → GC only → AChEi + GC	207 (7)
GC + NSIST → NSIST only → GC + NSIST	162 (5)
AChEi + GC → AChEi only → AChEi + GC	160 (5)
AChEi + GC + NSIST → AChEi + NSIST → AChEi + GC + NSIST	133 (4)

AChEi, acetylcholinesterase inhibitor; GC, glucocorticoid; IQR, interquartile range; NSIST, non-steroidal immunosuppressive therapy; SD, standard deviation.

### 3.3 Glucocorticoid exposure in patients with generalized myasthenia gravis

**Figure 3:**
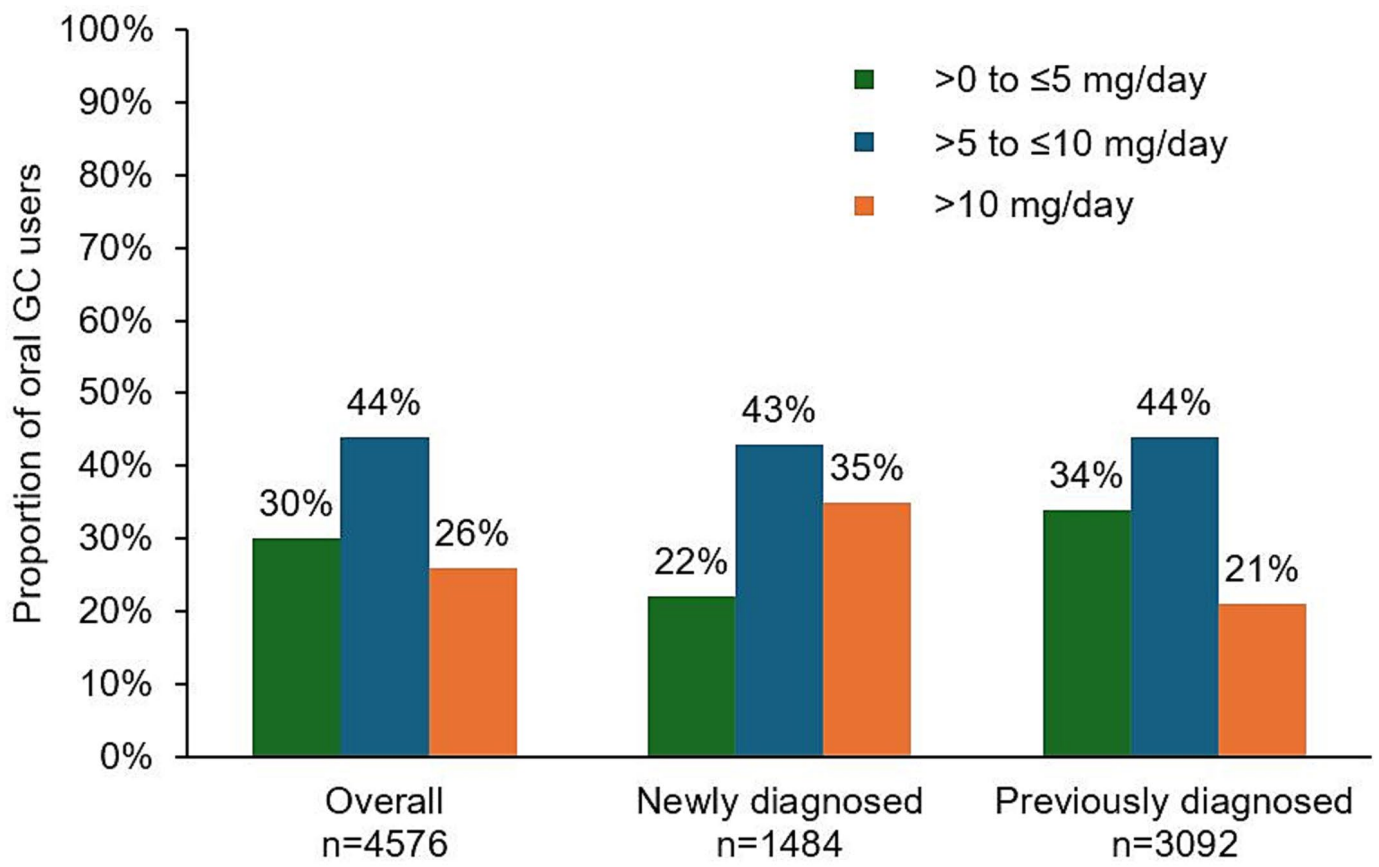
Distribution of oral GC average daily dose among patients with gMG who used at least one dose of oral GC. GC, glucocorticoid; gMG, generalized myasthenia gravis.

Nearly half of patients (47% [4,576/9687]) had records of oral GC treatment at least once at any time during the follow-up after index date (Table 3). Overall GC average daily dose (SD) among GC users was 8.5 (7.4) mg/day, with higher average daily dose observed among newly diagnosed patients compared with previously diagnosed patients (10.6 [10.0] mg/day and 7.5 [5.4] mg/day, respectively). Among those exposed to GC, 70% had an average daily dose exceeding the target of 5 mg/day (Figure 3), including 44% treated with >5 to ≤10 mg/day and 26% treated with daily doses exceeding 10 mg/day. A greater proportion of newly diagnosed patients received GC >10 mg/day compared with previously diagnosed patients (35 and 21%, respectively).

**Table 3:** Oral GC usage and dosing based on the exposed duration during the 5-year follow-up period among patients with gMG.

	Overall (*N* = 9,687)	Newly diagnosed (*n* = 3,696)	Previously diagnosed (*n* = 5,991)
**No oral GC**	5,111 (53)	2,212 (60)	2,899 (48)
**At least 1 dose of oral GC**	4,576 (47)	1,484 (40)	3,092 (52)
Daily dose (mg/day)			
Mean (SD)	8.5 (7.4)	10.6 (10.0)	7.5 (5.4)
Median (IQR)	6.6 (4.9–10.2)	8.2 (5.2–12.0)	5.9 (4.7–9.6)
Minimum	>0	>0	>0
Maximum	120	120	67

GC, glucocorticoid; gMG, generalized myasthenia gravis; IQR, interquartile range; SD, standard deviation.

### 3.4 Associations between glucocorticoid use and comorbidities

**Figure 4:**
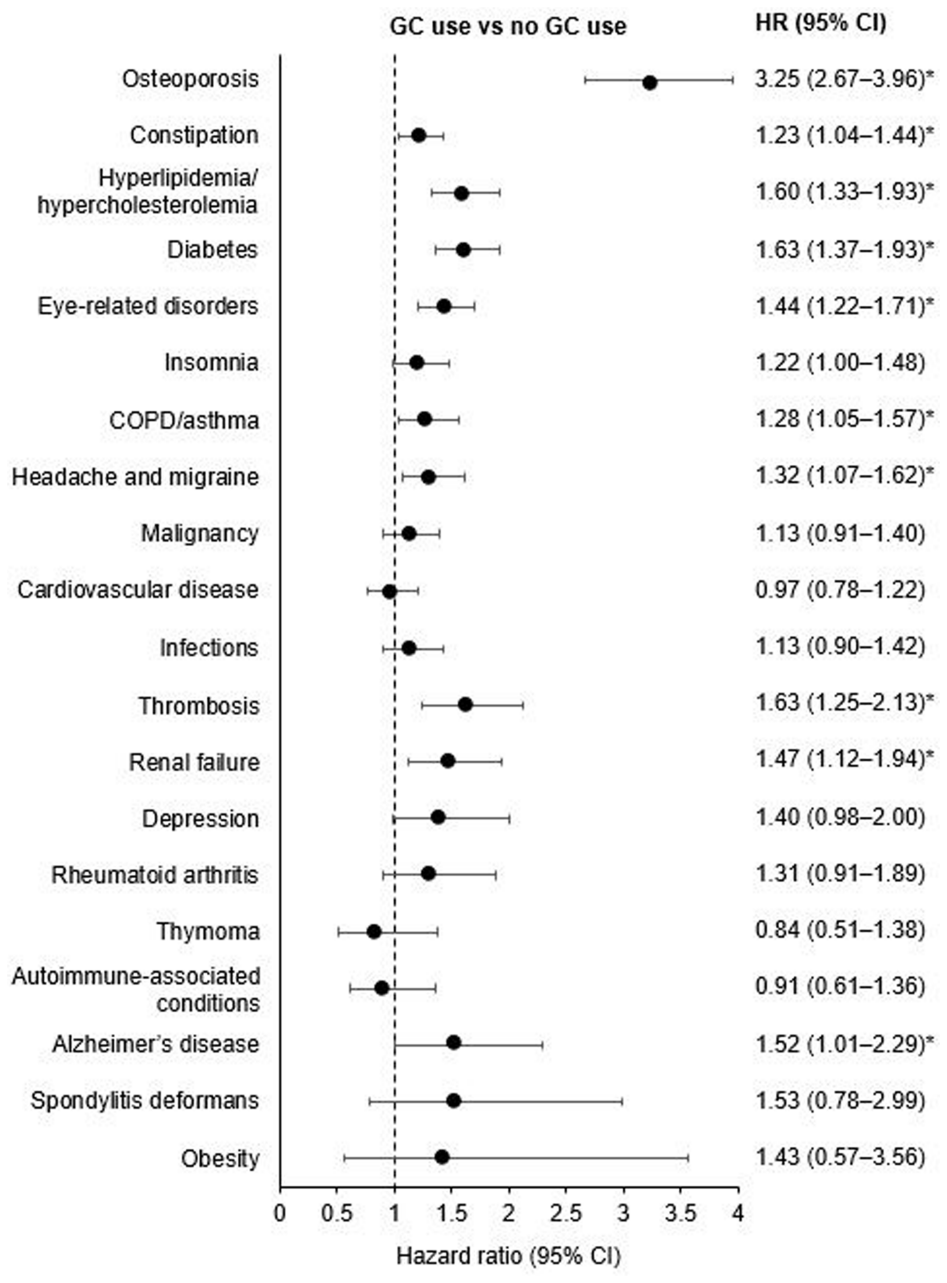
Associations between oral GC use and comorbidities in patients with gMG. *Denotes significance at a 95% confidence interval. CI, confidence interval; COPD, chronic obstructive pulmonary disease; GC, glucocorticoid; gMG, generalized myasthenia gravis; HR, hazard ratio.

The most frequently reported comorbidities among patients with gMG included osteoporosis, constipation, hyperlipidemia/hypercholesterolemia, diabetes, and eye-related disorders (Table 4). GC use was significantly associated with a range of comorbidities, most strongly with osteoporosis, diabetes, thrombosis, and hyperlipidemia/hypercholesterolemia (Figure 4). A dose-dependent relationship was observed for many conditions, with an increased risk of osteoporosis, hyperlipidemia/hypercholesterolemia, diabetes, and thrombosis evident even at low GC doses (≤5 mg/day) compared to patients with no GC exposure (Supplementary Table 3).

**Table 4:** Incidence of comorbidities among patients with gMG.

	Patients (*n*)	Events (*n*)	PY	Rate^a^
Osteoporosis	6,189	888	15,737	56.4
Constipation	7,172	993	21,131	47.0
Hyperlipidemia/hypercholesterolemia	6,525	747	17,935	41.7
Diabetes	7,298	877	21,182	41.4
Eye-related disorders (glaucoma, cataract, dry eye)	7,676	869	22,228	39.1
Insomnia	7,558	756	22,268	34.0
COPD/asthma	7,955	643	23,616	27.2
Headache and migraine	8,858	554	26,507	20.9
Malignancy	8,518	471	26,642	17.7
Cardiovascular diseases, CHF, CAD	8,984	474	27,789	17.1
Infections	9,276	429	28,556	15.0
Thrombosis	9,262	343	28,721	11.9
Renal failure	9,256	300	28,806	10.4
Depression	9,223	220	28,692	7.7
Rheumatoid arthritis	9,000	190	27,899	6.8
Thymoma	8,397	166	25,491	6.5
Autoimmune-associated conditions	9,124	150	28,435	5.3
Alzheimer’s disease	9,519	133	29,913	4.4
Spondylitis deformans	9,556	47	29,957	1.6
Obesity	9,631	35	30,241	1.2

^a^Number of events per 1,000 person-years of follow-up. CAD, coronary arterial disease; COPD, chronic obstructive pulmonary disease; CHF, chronic heart failure; FU, follow-up; gMG, generalized myasthenia gravis; PY, person-year.

The strongest association was found for osteoporosis (GC use vs. no GC use HR [95% CI]: 3.25 [2.67–3.96]). As osteoporosis codes may sometimes be used to prescribe preventive medications, a sensitivity test was performed using fracture codes. Results for fracture were consistent with those for osteoporosis, with a dose-dependent association demonstrated with GC use (HR [95% CI]: 1.53 [1.25–1.87]; Supplementary Figure 4).

## 4 Discussion

This large real-world study, utilizing the Japan MDV database, evaluated treatment patterns and the relationship between GC use and the incidence of comorbidities among patients with gMG, helping to address a critical evidence gap regarding the burden of gMG in Japan. The study findings demonstrated that the majority of patients with gMG in Japan were treated with AChEis, GCs, and NSISTs. Conversely, only a small proportion received other treatments such as IVIg, PLEX, biologics, or thymectomy. Notably, despite Japanese gMG treatment guidelines recommending low-dose GC therapy, many patients on GCs were prescribed doses exceeding the target daily dose of 5 mg prednisone equivalent. Furthermore, GC usage was associated with a significantly increased incidence of GC-associated comorbidities, in a dose-dependent manner.

Overall, the results were largely consistent with those observed in other geographies. The baseline characteristics in this cohort were comparable to those previously reported in real-world gMG populations in Japan, the US, and France (4, 25, 26, 29, 30). Similar to trends observed in the US and France, most patients with gMG in Japan were treated with monotherapy or combination therapies based on AChEis, GCs, and NSISTs (30, 31). Consistent with treatment patterns observed in the US (32), the usage of IVIg, PLEX, and biologics trended lower relative to standard-of-care treatments (AChEis, GCs, and NSISTs) throughout the study period. The low use of biologics during the study period may have been because eculizumab was the only biologic approved for gMG treatment in Japan at the index date for this study (33). It is also possible that these treatments were generally reserved for patients with severe or refractory disease. While approximately one-third of patients stayed on their initial regimen until the end of follow-up, some patients changed treatment combinations more frequently, which is consistent with treatment patterns observed in the US and other countries (14, 29, 31, 32, 34).

Differences in the observed treatment patterns between Japan and the US include a lower utilization of IVIg in Japan. Over the 5-year follow-up period, 8% of patients in the gMG cohort in Japan received IVIg, which is one-third lower than the approximately 11% of patients with gMG treated with IVIg in the US (35). This difference may be attributed to reduced availability of IVIg in Japan compared to the US, as well as additional treatment options such as IVMP or IAPP that may be used in Japan. Another important distinction is the widespread use of calcineurin inhibitors (CNIs) in Japanese practice. CNIs are often introduced as early-line immunosuppressive therapies in Japan (16). This reliance on potent oral NSISTs likely contributes to the relatively lower uptake of IVIg and newer biologics in Japan. While only 1.7% of patients in the gMG cohort in Japan were treated with IVIg during the first year post-index, IVIg usage stayed high during the 5-year follow-up period among frequent IVIg users, indicating a sustained treatment burden for this cohort. As more novel treatment options become available (15), this treatment burden (e.g., need for hospitalization for repeated infusions, invasiveness, and long infusion times) may be mitigated in the future by access to novel targeted therapies. However, there are currently no high-quality, head-to-head data comparing IVIg with newer biologics, and such agents also carry considerations of immunosuppression, cost, and access. Therefore, while biologics may offer alternative therapeutic approaches, further comparative studies are needed to establish their long-term benefit–risk profile compared with IVIg.

Long-term use of high-dose GCs is associated with a range of comorbidities, which has led to increasing efforts to reduce the use of high-dose GCs in patients with gMG (16, 36, 37). The recently revised gMG guidelines in Japan recommend achieving minimal manifestations of symptoms with an initial GC dose of up to 10 mg/day, followed by a reduction to ≤5 mg/day (18, 38), which is generally associated with a reduced risk of most GC-associated AEs and optimized HRQoL compared to higher GC doses (7, 34, 39, 40). In contrast, no equivalent target daily GC dose limit is provided in international gMG treatment guidelines (13, 14), and in the US, higher GC doses (even up to 100 mg/day or every other day) can be used as common therapeutic doses in clinical practice (41–43).

Despite these recommendations, our results suggest that GC use in Japan frequently exceeded the target daily dose of 5 mg in many patients with gMG and resulted in dose-dependent associations with the development of several GC-associated comorbidities. The higher GC dosing observed among newly diagnosed patients compared with previously diagnosed patients suggests that GC dosing may be reduced during the course of gMG treatment, aligned with treatment guidance. However, our results still underscore a persistent reliance on GCs for gMG management in Japan, as over one-third of newly diagnosed patients were treated with GCs >10 mg/day, which exceed the recommended initial GC dose of ≤10 mg/day. The significant association of GC use, even at doses below 5 mg/day, with several GC-associated adverse effects corroborates previous studies reporting GC-associated AEs across dosing levels despite the common preconception that adverse effects are generally more common in patients taking high doses of GCs (i.e., >30 mg/day) (41). As GC-associated AEs negatively impact HRQoL in patients with gMG (7, 44), tapering or eliminating long-term use of GC may be critical to holistically reducing the treatment burden in people living with gMG.

More than half of the patients in this study were elderly and female and therefore at high risk of comorbidities such as osteoporosis and fractures (45), meaning that their GC exposure ought to be minimized to avoid further increasing the risk of osteoporosis and fractures. However, Japan MG registry surveys carried out in 2012, 2015, and 2021 observed no change in daily GC dose for gMG over a 10-year follow-up period (2012: 4.5 mg/day; 2015: 4.6 mg/day; 2021: 4.5 mg/day), suggesting that GC use for gMG in Japan remained relatively constant between 2012 and 2021 (46). This study emphasizes the importance of continuing to promote the use of low-dose GCs (i.e., up to 5 mg per day) in Japan and suggests that other countries should consider adopting similar strategies to aggressively reduce GC exposure for patients with gMG. For osteoporosis, diabetes, thrombosis, and hyperlipidemia/hypercholesterolemia, significant associations with GC use were observed even at doses up to 5 mg/day, indicating that GC use should ideally be reduced wherever possible. Alternative steroid-sparing gMG therapies, including biologics, should be proactively considered in Japan to help every patient achieve the ambitious treatment goal of minimal disease manifestations with oral GCs at doses of up to 5 mg/day.

Limitations of this study include that the results may not be representative of all patients with gMG in Japan, as data were captured exclusively from the MDV dataset comprising information from a network of hospitals; thus, data from services that occur outside of the network are not captured, which may affect outcome and exposure assessments. Additionally, the Japan MDV dataset is observational in nature and collected for administrative purposes, which limits causal inference in analyses comparing different gMG treatments. As an administrative dataset, MDV may lack key clinical information, further restricting the generalizability of our findings. Another limitation of utilizing the Japan MDV dataset in the present study was the inability to capture the exact duration of high-dose exposure and tapering schedules; short-term higher-dose regimens may have a different benefit–risk profile compared with prolonged exposure at similar doses. Moreover, given the relatively low mean daily GC dose observed in this study and the predominance of older patients, it is difficult to attribute all recorded comorbidities solely to GC exposure. Future prospective studies, ideally incorporating clinical registries or interventional trials, are warranted to validate the benefit–risk profile of GC use in patients with gMG in Japan. Furthermore, GC exposure was calculated based on the first year of GC usage only, and a longer follow-up period may be required for analyses on long-term effects of cumulative GC exposure in patients with gMG.

Patient characteristics and treatment patterns among patients with gMG in Japan were consistent with expectations and the published literature and similar to those observed in other global regions, with most patients using AChEi, GC, or NSIST monotherapy or combination therapies. Despite specific local guidelines targeting limited steroid exposure (up to 5 mg/day), many patients in Japan received GCs exceeding 5 mg/day. Compared to those not treated with GCs, exposure to GCs was associated with higher rates of several comorbidities including osteoporosis, diabetes, thrombosis, and hyperlipidemia/hypercholesterolemia, in a dose-dependent manner. To achieve treatment goals, patients with gMG in Japan and across all global regions may benefit from alternative treatment approaches to reduce or eliminate GC usage.

## Data Availability

The data analyzed in this study is subject to the following licenses/restrictions: Anonymized data were derived from the Medical Data Vision (MDV) database in Japan. Requests to access these datasets should be directed to https://en.mdv.co.jp/.
