# Supplementary material for: Treatment patterns and impact of glucocorticoids on health outcomes in generalized myasthenia gravis: a retrospective observational study based on the Medical Data Vision database in Japan
Source: Front Neurol. 2025 Oct 10;16:1625457. doi: 10.3389/fneur.2025.1625457 (PMC12550776; doi:10.3389/fneur.2025.1625457)
Supplement: Supplementary file 1 [file Supplementary_file_1.docx]

Supplementary Material

# Supplementary tables

## Supplementary Table 1. Exclusionary diagnoses.

| Disease name | ICD-10 code | Disease code |
| --- | --- | --- |
| Chronic inflammatory demyelinating polyneuropathy (CIDP) | G61.8 | 8841670 |
| Polymyositis | M33.2 | 7104004 |
| Multiple sclerosis | G35.0 | 3409005 |
| Neuromyelitis optica | G36.0 | 3410003 |
| Guillain–Barré syndrome (GBS) | G61.0 | 3570001 |
| Dermatomyositis | M33.9 | 7103007 |
| Myelitis | G04.9 | 3239013 |
| Miller Fisher syndrome | G61.0 | 8847257 |
| Encephalitis | G04.9 | 3239015 |
| Meningoencephalitis | G04.9 | 8835801 |
| Fisher syndrome | G61.0 | 3570002 |
| Sporadic inclusion body myositis | M33.2 | 7104007 |
| Acute myelitis | G04.9 | 3239007 |
| Acute multiple sclerosis | G35.0 | 8832406 |
| Spinal multiple sclerosis | G35.0 | 8835995 |
| Opticospinal multiple sclerosis | G36.0 | 8846138 |
| Encephalomyelitis | G04.9 | 8838733 |
| Chronic encephalitis | G04.9 | 8840383 |
| Amyotrophic lateral sclerosis | G12.2 | 3352007 |
| Mitochondrial encephalomyopathy | G71.3 | 8841410 |
| Ophthalmoplegic migraine | G43.8 | 3468001 |
| LEMS (Lambert-Eaton myasthenic syndrome) | C80.9 | 8841315 |
| MMN (multifocal motor neuropathy) | G61.8 | 8841400 |
| Limbic encephalitis | G04.9 | 8844116 |

## Supplementary Table 2. Overall treatment utilization over follow-up for various cohorts.

1. **All patients with gMG (N=9687)**

|  | **Baseline** (pre-index year) | **Year 1** | **Year 2** | **Year 3** | **Year 4** | **Year 5** | **Overall** |
| --- | --- | --- | --- | --- | --- | --- | --- |
| **All patients with at least 1 claim activity, n** | **9687** | **9687** | **8078** | **6642** | **5550** | **4386** | **9687** |
| **No gMG treatment data in the dataset, n (%)** | 4511 (47) | 2801 (29) | 2740 (34) | 2195 (33) | 1824 (33) | 1321 (30) | 2542 (26) |
| **Patient with at least one claim for gMG related treatments, n (%)^a^** | 5176 (53) | 6886 (71) | 5338 (66) | 4447 (67) | 3726 (67) | 3065 (70) | 7145 (74) |
| **AChEi** | 3135 (32) | 4420 (46) | 3147 (39) | 2557 (38) | 2086 (38) | 1688 (38) | 4719 (49) |
| **Oral GCs** | 3258 (34) | 4173 (43) | 3518 (44) | 2933 (44) | 2466 (44) | 2041 (47) | 4576 (47) |
| **IVMP** | 271 (3) | 460 (5) | 196 (2) | 138 (2) | 112 (2) | 92 (2) | 697 (7) |
| **NSISTs** | 2169 (22) | 2964 (31) | 2599 (32) | 2209 (33) | 1898 (34) | 1600 (36) | 3266 (34) |
| **IVIg** | 307 (3) | 534 (6) | 256 (3) | 217 (3) | 159 (3) | 138 (3) | 798 (8) |
| **PLEX** | 93 (1) | 142 (1) | 65 (1) | 45 (1) | 30 (1) | 21 (0) | 214 (2) |
| **Biologics** | 13 (0) | 36 (0) | 33 (0) | 43 (1) | 39 (1) | 61 (1) | 120 (1) |
| **Thymectomy** | 68 (1) | 105 (1) | 7 (0) | 1 (0) | 1 (0) | 1 (0) | 115 (1) |

^a^Percentages may not add up to 100% as patients may have multiple treatments.

AChEi, acetylcholinesterase inhibitor; GC, glucocorticoid; gMG, generalized myasthenia gravis; IVMP, intravenous methylprednisone; IVIg, intravenous immunoglobulin; IVMP, intravenous methylprednisolone; NSIST, non-steroidal immunosuppressive therapy; PLEX, plasma exchange.

1. **Newly diagnosed patients with gMG (n=3696)**

|  | **Baseline** (pre-index year) | **Year 1** | **Year 2** | **Year 3** | **Year 4** | **Year 5** | **Overall** |
| --- | --- | --- | --- | --- | --- | --- | --- |
| **Newly diagnosed patients with at least 1 claim activity, n** | 3696 | 3696 | 2668 | 1702 | 994 | 396 | 3696 |
| **No gMG treatment data in the dataset, n (%)** | 2971 (80) | 1159 (31) | 1266 (47) | 832 (49) | 502 (51) | 204 (52) | 1086 (29) |
| **Patient with at least one claim for gMG related treatments, n (%)^a^** | 725 (20) | 2537 (69) | 1402 (53) | 870 (51) | 492 (49) | 192 (48) | 2610 (71) |
| **AChEi** | 262 (7) | 1762 (48) | 794 (30) | 475 (28) | 249 (25) | 93 (23) | 1807 (49) |
| **Oral GCs** | 438 (12) | 1,353 (37) | 919 (34) | 568 (33) | 320 (32) | 128 (32) | 1484 (40) |
| **IVMP** | 64 (2) | 295 (8) | 53 (2) | 35 (2) | 23 (2) | 4 (1) | 349 (9) |
| **NSISTs** | 190 (5) | 891 (24) | 648 (24) | 405 (24) | 237 (24) | 102 (26) | 978 (26) |
| **IVIg** | 11 (0) | 19 (1) | 8 (0) | 13 (1) | 6 (1) | 2 (1) | 37 (1) |
| **PLEX** | 66 (2) | 325 (9) | 76 (3) | 49 (3) | 27 (3) | 7 (2) | 373 (10) |
| **Biologics** | 2 (0) | 79 (2) | 14 (1) | 7 (0) | 2 (0) | 1 (0) | 91 (2) |
| **Thymectomy** | 5 (0) | 93 (3) | 2 (0) | 1 (0) | 1 (0) | 0 (0) | 97 (3) |

^a^Percentages may not add up to 100% as patients may have multiple treatments.

AChEi, acetylcholinesterase inhibitor; GC, glucocorticoid; gMG, generalized myasthenia gravis; IVMP, intravenous methylprednisone; IVIg, intravenous immunoglobulin; IVMP, intravenous methylprednisolone; NSIST, non-steroidal immunosuppressive therapy; PLEX, plasma exchange.

1. **Previously diagnosed patients with gMG (n=5991)**

|  | **Baseline** (pre-index year) | **Year 1** | **Year 2** | **Year 3** | **Year 4** | **Year 5** | **Overall** |
| --- | --- | --- | --- | --- | --- | --- | --- |
| **Previously diagnosed patients with at least 1 claim activity, n** | 5991 | 5991 | 5410 | 4940 | 4556 | 3990 | 5991 |
| **No gMG treatment data in the dataset, n (%)** | 1540 (26) | 1642 (27) | 1496 (28) | 1379 (28) | 1321 (29) | 1115 (28) | 1456 (24) |
| **Patient with at least one claim for gMG related treatments, n (%)^a^** | 4451 (74) | 4348 (73) | 3913 (72) | 3560 (72) | 3234 (71) | 2873 (72) | 4534 (76) |
| **AChEi** | 2873 (48) | 2658 (44) | 2353 (43) | 2081 (42) | 1837 (40) | 1595 (40) | 2912 (49) |
| **Oral GCs** | 2820 (47) | 2819 (47) | 2599 (48) | 2364 (48) | 2146 (47) | 1913 (48) | 3091 (52) |
| **IVMP** | 207 (3) | 165 (3) | 143 (3) | 103 (2) | 89 (2) | 88 (2) | 348 (6) |
| **NSISTs** | 1979 (33) | 2072 (35) | 1951 (36) | 1803 (36) | 1661 (36) | 1498 (38) | 2287 (38) |
| **IVIg** | 2 (0) | 17 (0) | 25 (0) | 30 (1) | 33 (1) | 59 (1) | 83 (1) |
| **PLEX** | 241 (4) | 209 (3) | 180 (3) | 168 (3) | 132 (3) | 131 (3) | 425 (7) |
| **Biologics** | 91 (2) | 63 (1) | 51 (1) | 38 (1) | 28 (1) | 20 (1) | 123 (2) |
| **Thymectomy** | 63 (1) | 12 (0) | 5 (0) | 0 (0) | 0 (0) | 1 (0) | 18 (0) |

^a^Percentages may not add up to 100% as patients may have multiple treatments.

AChEi, acetylcholinesterase inhibitor; GC, glucocorticoid; gMG, generalized myasthenia gravis; IVMP, intravenous methylprednisone; IVIg, intravenous immunoglobulin; IVMP, intravenous methylprednisolone; NSIST, non-steroidal immunosuppressive therapy; PLEX, plasma exchange.

1. **Patients with chronic IVIg usage in Year 1 (n=167)**

|  | **Year 1** | **Year 2** | **Year 3** | **Year 4** | **Year 5** | **Overall** |
| --- | --- | --- | --- | --- | --- | --- |
| **Patient with at least 1 claim activity, n** | 167 | 146 | 120 | 85 | 56 | 167 |
| **No gMG treatment data in the dataset, n (%)** | 0 (0) | 4 (3) | 7 (6) | 2 (2) | 1 (2) | 0 (0) |
| **Patient with at least one claim for gMG related treatments, n (%)^a^** | **167 (100)** | **142 (97)** | **113 (94)** | **83 (98)** | **55 (98)** | **167 (100)** |
| **Oral GCs** | 144 (86) | 128 (88) | 104 (87) | 75 (88) | 48 (86) | 148 (89) |
| **IVMP** | 62 (37) | 36 (25) | 24 (20) | 21 (25) | 8 (14) | 73 (44) |
| **NSISTs** | 142 (85) | 113 (77) | 91 (76) | 71 (84) | 47 (84) | 146 (87) |
| **AChEi** | 84 (50) | 76 (52) | 63 (53) | 47 (55) | 34 (61) | 89 (53) |
| **Biologics** | 6 (4) | 13 (9) | 17 (14) | 15 (18) | 10 (18) | 26 (16) |
| **IVIg** | 167 (100) | 101 (69) | 76 (63) | 51 (60) | 33 (59) | 167 (100) |
| **PLEX** | 32 (19) | 20 (14) | 17 (14) | 10 (12) | 6 (11) | 44 (26) |
| **Thymectomy** | 2 (1) | 0 (0) | 0 (0) | 0 (0) | 0 (0) | 2 (1) |

^a^Percentages may not add up to 100% as patients may have multiple treatments.

AChEi, acetylcholinesterase inhibitor; GC, glucocorticoid; gMG, generalized myasthenia gravis; IVMP, intravenous methylprednisone; IVIg, intravenous immunoglobulin; IVMP, intravenous methylprednisolone; NSIST, non-steroidal immunosuppressive therapy; PLEX, plasma exchange.

## Supplementary Table 3. Associations between oral GC use and comorbidities in patients with gMG.

|  | **No GC** | **GC** | **GC vs no GC** | | | |
| --- | --- | --- | --- | --- | --- | --- |
|  |  |  | **Overall** | **Low-dose GC^a^ vs no GC** | **Medium-dose GC^b^ vs no GC** | **High-dose GC^c^  vs no GC** |
| **Comorbidity** | **Rate (events/ 1000 PY of FU)** | | **HR (95% CI)** | | | |
| Osteoporosis | 20.3 | 75.1 | 3.25 (2.67–3.96)* | 2.56 (2.00–3.29)* | 3.71 (2.83–4.84)* | 5.19 (3.81–7.09)* |
| Constipation | 31.1 | 35.6 | 1.23 (1.04–1.44)* | 1.23 (1.00–1.50) | 1.10 (0.88–1.37) | 1.52 (1.17–1.97)* |
| Hyperlipidemia/ hypercholesterolemia | 24.7 | 37.8 | 1.60 (1.33–1.93)* | 1.38 (1.08–1.76)* | 1.47 (1.14–1.90)* | 2.44 (1.86–3.20)* |
| Diabetes | 23.3 | 39.3 | 1.63 (1.37–1.93)* | 1.38 (1.11–1.71)* | 1.74 (1.40–2.16)* | 2.10 (1.63–2.71)* |
| Eye-related disorders | 24.9 | 34.1 | 1.44 (1.22–1.71)* | 1.23 (0.99–1.53) | 1.40 (1.12–1.75)* | 2.17 (1.69–2.78)* |
| Insomnia | 19.9 | 26.2 | 1.22 (1.00–1.48) | 0.98 (0.75–1.27) | 1.32 (1.03–1.69)* | 1.62 (1.21–2.18)* |
| COPD/asthma | 17.3 | 21.8 | 1.28 (1.05–1.57)* | 1.17 (0.90–1.52) | 1.45 (1.12–1.88)* | 1.22 (0.87–1.71) |
| Headache and migraine | 14.1 | 20.7 | 1.32 (1.07–1.62)* | 1.10 (0.83–1.44) | 1.40 (1.08–1.81)* | 1.65 (1.23–2.22)* |
| Malignancy | 13.7 | 16.1 | 1.13 (0.91-1.40) | 1.23 (0.95-1.60) | 1.06 (0.80-1.40) | 1.05 (0.73-1.51) |
| Cardiovascular disease | 13.7 | 12.5 | 0.97 (0.78-1.22) | 0.87 (0.65-1.16) | 1.01 (0.75-1.36) | 1.24 (0.84-1.82) |
| Infections | 11.9 | 13.2 | 1.13 (0.90–1.42) | 1.08 (0.81–1.44) | 1.03 (0.76–1.41) | 1.50 (1.05–2.14)* |
| Thrombosis | 7 | 12.2 | 1.63 (1.25–2.13)* | 1.41 (1.01–1.97)* | 1.51 (1.07–2.13)* | 2.60 (1.78–3.79)* |
| Renal failure | 6.6 | 10.9 | 1.47 (1.12–1.94)* | 1.34 (0.95–1.88) | 1.54 (1.09–2.17)* | 1.70 (1.11–2.61)* |
| Depression | 4.5 | 6.6 | 1.40 (0.98–2.00) | 1.18 (0.75–1.87) | 1.35 (0.85–2.14) | 2.03 (1.24–3.32)* |
| Rheumatoid arthritis | 4.3 | 6.1 | 1.31 (0.91-1.89) | 1.21 (0.76-1.92) | 1.33 (0.84-2.12) | 1.53 (0.88-2.67) |
| Thymoma | 2.8 | 3.5 | 0.84 (0.51-1.38) | 1.06 (0.59-1.90) | 0.64 (0.32-1.28) | 0.78 (0.37-1.66) |
| Autoimmune-associated conditions | 4.2 | 4.2 | 0.91 (0.61-1.36) | 0.74 (0.42-1.30) | 0.93 (0.54-1.58) | 1.24 (0.68-2.24) |
| Alzheimer’s disease | 3.1 | 4 | 1.52 (1.01–2.29)* | 1.36 (0.82–2.28) | 1.58 (0.93–2.67) | 1.93 (0.96–3.88) |
| Spondylitis deformans | 1.2 | 1.4 | 1.53 (0.78-2.99) | 1.39 (0.60-3.24) | 1.24 (0.48-3.20) | 2.67 (0.99-7.18) |
| Obesity | 0.6 | 0.9 | 1.43 (0.57-3.56) | 1.19 (0.36-3.92) | 1.58 (0.50-4.96) | 1.70 (0.46-6.36) |

^a^≤5 mg/day GC equivalent of prednisone. ^b^>5 to ≤10 mg/day GC equivalent of prednisone. ^c^>10 mg/day GC equivalent of prednisone.

*Denotes significance at a 95% confidence interval.

CI, confidence interval; COPD, chronic obstructive pulmonary disease; FU, follow-up; GC, glucocorticoid; HR, hazard ratio; gMG, generalized myasthenia gravis; PY, person-years.

# Supplementary figures

## Supplementary Figure 1. Study design overview.


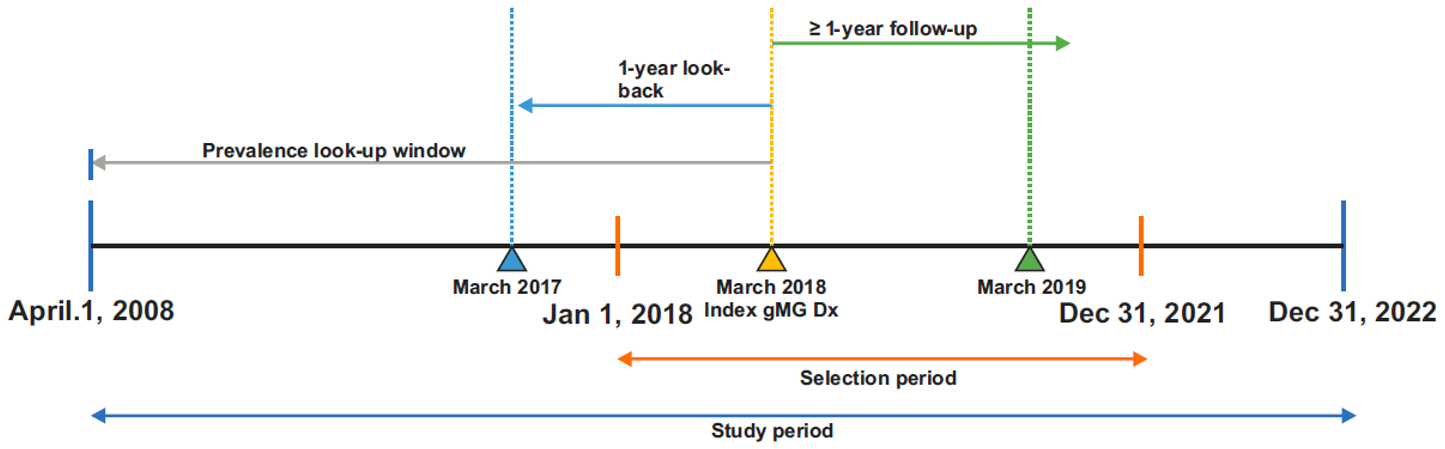


Dx, diagnosis; gMG, generalized myasthenia gravis.

## Supplementary Figure 2. Determination of the average daily dose of GC (prednisone equivalent).


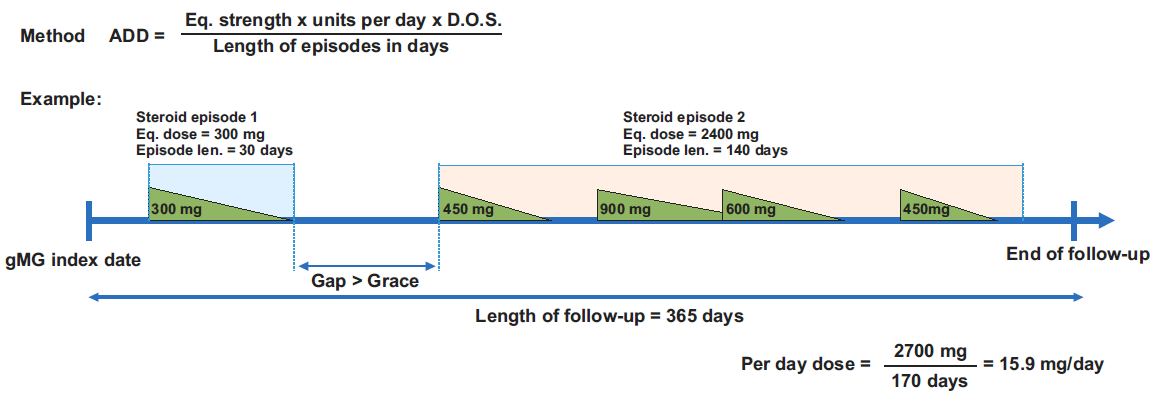
ADD, average daily dose; D.O.S., days of supply; GC, glucocorticoid; gMG, generalized myasthenia gravis.

## Supplementary Figure 3. gMG cohort treatment patterns in the overall cohort (A), in newly diagnosed patients (B), and in previously diagnosed patients (C).

L1, first line; L2, second line; L3, third line; AChEi, acetylcholinesterase inhibitor; gMG, generalized myasthenia gravis; NSIST, non-steroidal immunosuppressive therapy; SoC, standard of care.


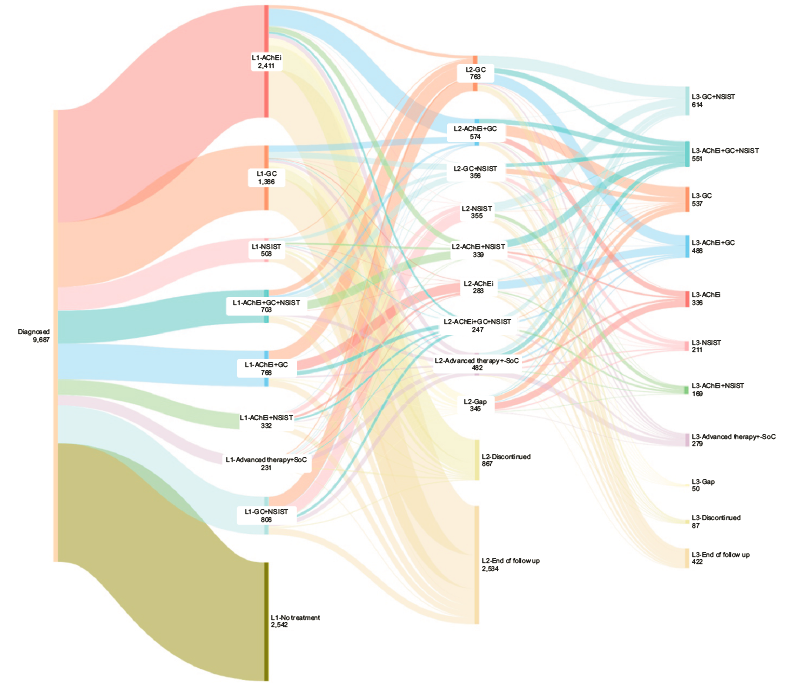
**A) Treatment patterns in the full cohort.**


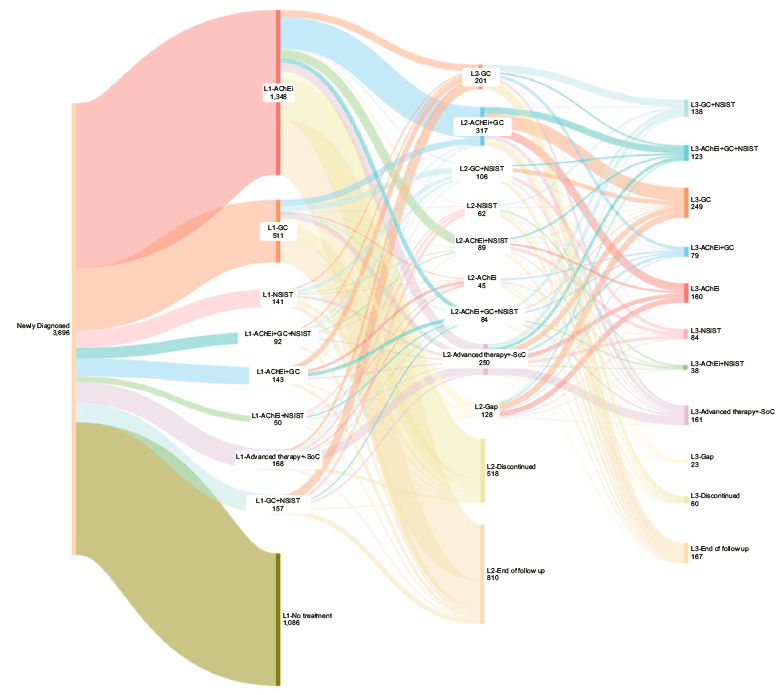
**B) Treatment patterns in newly diagnosed patients.**


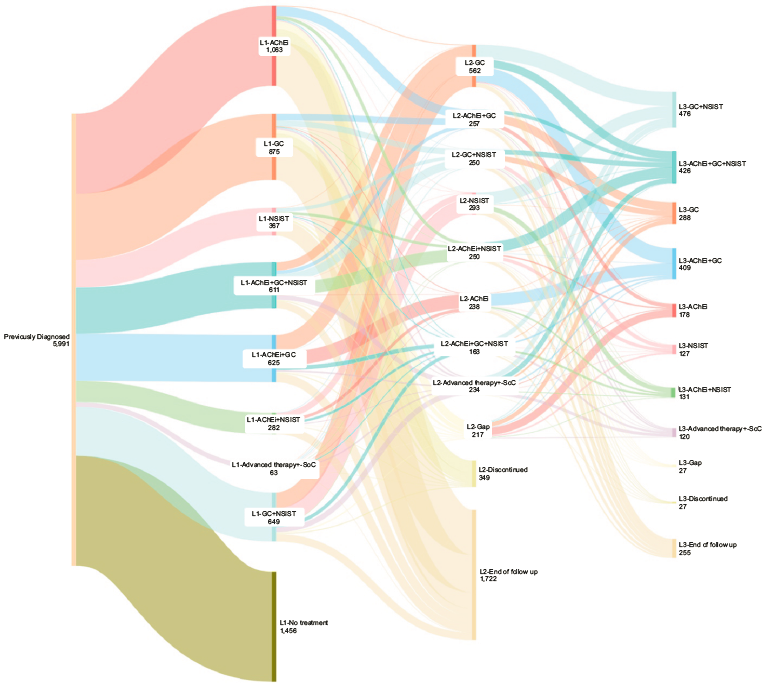
**C) Treatment patterns in previously diagnosed patients.**

##
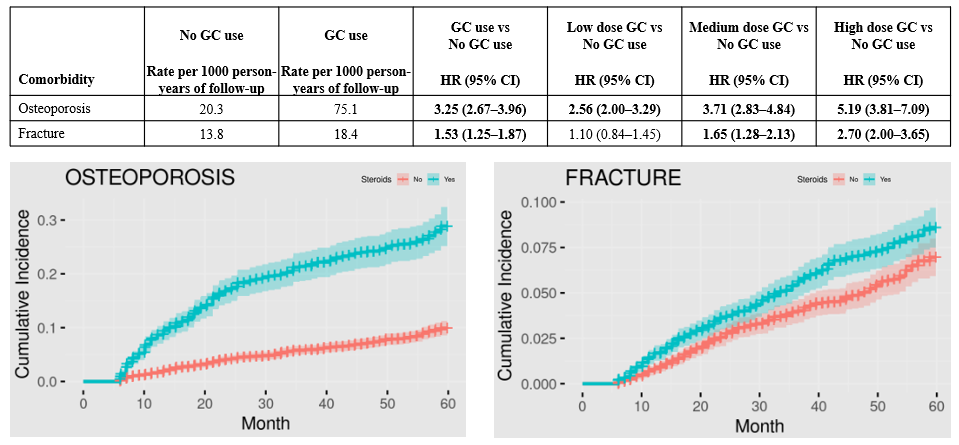
Supplementary Figure 4. Association between glucocorticoid use and occurrence of osteoporosis and fractures.

|  | **No GC use** | **GC use** | **GC use vs No GC use** | **Low-dose GC vs No GC use** | **Medium-dose GC vs No GC use** | **High-dose GC vs No GC use** |
| --- | --- | --- | --- | --- | --- | --- |
| **Comorbidity** | **Rate per 1000 person-years of follow-up** | **Rate per 1000 person-years of follow-up** | **HR (95% CI)** | **HR (95% CI)** | **HR (95% CI)** | **HR (95% CI)** |
| Osteoporosis | 20.3 | 75.1 | 3.25 (2.67–3.96) | 2.56 (2.00–3.29) | 3.71 (2.83–4.84) | 5.19 (3.81–7.09) |
| Fracture | 13.8 | 18.4 | 1.53 (1.25–1.87) | 1.10 (0.84–1.45) | 1.65 (1.28–2.13) | 2.70 (2.00–3.65) |

CI, confidence interval; GC, glucocorticoid; HR, hazard ratio.
